# Effects of Inner Nuclear Membrane Proteins SUN1/UNC-84A and SUN2/UNC-84B on the Early Steps of HIV-1 Infection

**DOI:** 10.1128/JVI.00463-17

**Published:** 2017-09-12

**Authors:** Torsten Schaller, Lorenzo Bulli, Darja Pollpeter, Gilberto Betancor, Juliane Kutzner, Luis Apolonia, Nikolas Herold, Robin Burk, Michael H. Malim

**Affiliations:** a Department of Infectious Diseases, Virology, University Hospital Heidelberg, Heidelberg, Germany; b Department of Infectious Diseases, King's College London, London, United Kingdom; c Institute for Medical Virology, Goethe University, Frankfurt am Main, Germany; d Childhood Cancer Research Unit, Astrid Lindgren Children's Hospital, Karolinska Hospital, Stockholm, Sweden; e Department of Children's and Women's Health, Astrid Lindgren Children's Hospital, Karolinska University Hospital, Stockholm, Sweden; University of Utah

**Keywords:** CA, HIV-1, SUN1, SUN2, cyclophilin A, early infection, nuclear envelope, nuclear import, nuclear pore complex, transmitted founder virus

## Abstract

Human immunodeficiency virus type 1 (HIV-1) infection of dividing and nondividing cells involves regulatory interactions with the nuclear pore complex (NPC), followed by translocation to the nucleus and preferential integration into genomic areas in proximity to the inner nuclear membrane (INM). To identify host proteins that may contribute to these processes, we performed an overexpression screen of known membrane-associated NE proteins. We found that the integral transmembrane proteins SUN1/UNC84A and SUN2/UNC84B are potent or modest inhibitors of HIV-1 infection, respectively, and that suppression corresponds to defects in the accumulation of viral cDNA in the nucleus. While laboratory strains (HIV-1_NL4.3_ and HIV-1_IIIB_) are sensitive to SUN1-mediated inhibition, the transmitted founder viruses RHPA and ZM247 are largely resistant. Using chimeric viruses, we identified the HIV-1 capsid (CA) protein as a major determinant of sensitivity to SUN1, and *in vitro*-assembled capsid-nucleocapsid (CANC) nanotubes captured SUN1 and SUN2 from cell lysates. Finally, we generated *SUN1*^−/−^ and *SUN2*^−/−^ cells by using CRISPR/Cas9 and found that the loss of SUN1 had no effect on HIV-1 infectivity, whereas the loss of SUN2 had a modest suppressive effect. Taken together, these observations suggest that SUN1 and SUN2 may function redundantly to modulate postentry, nuclear-associated steps of HIV-1 infection.

**IMPORTANCE** HIV-1 causes more than 1 million deaths per year. The life cycle of HIV-1 has been studied extensively, yet important steps that occur between viral capsid release into the cytoplasm and the expression of viral genes remain elusive. We propose here that the INM components SUN1 and SUN2, two members of the linker of nucleoskeleton and cytoskeleton (LINC) complex, may interact with incoming HIV-1 replication complexes and affect key steps of infection. While overexpression of these proteins reduces HIV-1 infection, disruption of the individual *SUN2* and *SUN1* genes leads to a mild reduction or no effect on infectivity, respectively. We speculate that SUN1/SUN2 may function redundantly in early HIV-1 infection steps and therefore influence HIV-1 replication and pathogenesis.

## INTRODUCTION

The nuclear envelope (NE) consists of the nuclear lamina and the nuclear membrane, comprising the INM, the perinuclear space (PNS), and the outer nuclear membrane (ONM), a continuum of the endoplasmic reticulum that spatially separates the nucleoplasm from the cytoplasm. The NE serves as a platform for the assembly of large protein scaffolds, such as the nuclear pore complexes (NPCs) that allow the transport of proteins, nucleic acids, and other molecules between the cytoplasm and nucleus. HIV-1 engages components of the NPC to enable nuclear import and interactions between HIV-1 CA and the NPC proteins NUP153 (1–4) and NUP358 (3, 5–8), as well as with the shuttling protein cleavage and polyadenylation specific factor 6 (CPSF6) (9–11), and these components may play important roles in this process. Following nuclear localization, HIV-1 DNA integrates into transcriptionally active chromatin areas, preferentially in proximity to the NE (12), forming the provirus. The HIV-1 integrase (IN)-interacting lens epithelium-derived growth factor (LEDGF/p75) (13, 14) and the CA-interacting CPSF6 (9, 11) also contribute to HIV-1 integration (15–18). However, the molecular details of how HIV-1 targets preferred sites for provirus establishment and what the consequences of site selection are for virus replication or latency, as well as the identity and functions of other participating host proteins, are largely unknown.

Hundreds of proteins have been identified in enriched NE preparations, yet the functions of many of them are incompletely understood (19). Included among these are proteins that comprise the LINC complex, which physically connects proteins that constitute the nuclear skeleton (e.g., nuclear lamins) with proteins of the cytoskeleton (e.g., actin, microtubules, and intermediate filaments) (20). Here, we describe an ectopic expression screen to identify membrane-associated NE proteins that impact HIV-1 infection. Overexpression of the INM protein Sad1/UNC-84 domain-containing 2 (SUN2) has recently been described to inhibit HIV-1 infection (21, 22), and we asked whether other proteins exert a similar activity. In addition to SUN2, we found that overexpression of SUN1 strongly inhibited HIV-1 in a CA-specific manner.

Mammalian SUN1 and SUN2 were first identified as homologues of the Caenorhabditis elegans proteins UNC-84A and UNC-84B, respectively, and share a conserved carboxy-terminal SUN domain that is also found in Sad1 in Saccharomyces cerevisiae (23). Both proteins interact via their amino-terminal domains with proteins in the nuclear lamina, such as lamin A (LMNA) (24, 25) and emerin (EMD) (26), and in the PNS via their carboxy-terminal SUN domains with the Klarsicht-ANC1-Syne-homology (KASH) domains of nesprin proteins anchored in the ONM (23–25, 27–31). They are therefore important for several cellular processes, including telomere attachment to the NE in meiosis and for postmitotic cells (32–36), the DNA damage response (DDR) (37, 38), pre-double-strand break (DSB) and post-DSB homologue pairing (37), the removal of membranes from chromatin during mitosis (38), positioning of the nucleus, and cell migration and polarization (23, 39–43). Intriguingly, the greater mobility of damaged chromatin requires SUN1 and SUN2, since gene knockout of both in murine cells reduced the mobility of DNA damage foci (44). For SUN1, many splice isoforms have been described, and some are expressed in a cell-type-dependent manner (45). In addition, SUN1 can form homodimers or heterodimers with SUN2 (46) and associates with components of the NPC (47).

Genes encoding NE proteins have been implicated in a variety of inherited disorders affecting muscle, bones, neurons, and adipose tissue (48). SUN1 and SUN2 gene dysfunction has been associated with cardiomyopathies and skeletal myopathies (26, 49) and has been observed in certain cancer tissues, suggesting possible tumor suppressor activity (50, 51). While specific *SUN1* and *SUN2* alleles have been directly connected to Emery-Dreifuss muscular dystrophy (EDMD) and related myopathies (49), SUN proteins may also have indirect effects in disorders in which the *LMNA* gene is mutated. In cells from *LMNA*-deficient mice, which represent a model for EMDM and the premature aging Hutchinson-Gilford progeria syndrome (HGPS), SUN1 accumulates to abnormal levels (52). Surprisingly, mice that are also *SUN1* deficient show dramatically reduced pathological effects associated with the *LMNA* deficiency, suggesting that elevated SUN1 levels may contribute to the pathological phenotype, by an unknown mechanism(s) (52). Whether the functions of SUN1 and SUN2 in the DDR are connected with their roles in myopathies and other laminopathies is currently unresolved.

We demonstrate here that, similar to SUN2 (21), overexpression of SUN1 blocks nuclear import of HIV-1. HIV-2_ROD_ was also sensitive to SUN1 overexpression; however, other lentiviruses, such as simian immunodeficiency virus from macaque (SIV_mac_), were insensitive. Using chimeric viruses, we mapped the sensitivity determinant to the HIV-1 CA protein. We found that both SUN1 and SUN2 can interact with *in vitro*-assembled HIV-1 capsid-nucleocapsid (CANC) nanotubes, potentially reflecting a direct interaction with CA. Mutational analysis demonstrated that the amino-terminal domain of SUN1 is important for the observed block of HIV-1 infection. While overexpression of SUN1 or SUN2 blocked HIV-1 infection, gene disruption in THP-1 cells had no (SUN1) or modest (SUN2) effects on HIV-1 infectivity, possibly indicating redundancy between the two proteins. We did not observe a connection of endogenous SUN1 or SUN2 expression with cyclophilin A (CypA)-mediated activities or processes during early HIV-1 infection steps, as has been proposed for SUN2 (53), suggesting that such effects may be cell type and/or context specific.

## RESULTS

### An overexpression screen identified the inner nuclear membrane proteins SUN1 and SUN2 as potential effectors of HIV-1 infection.

**FIG 1:**
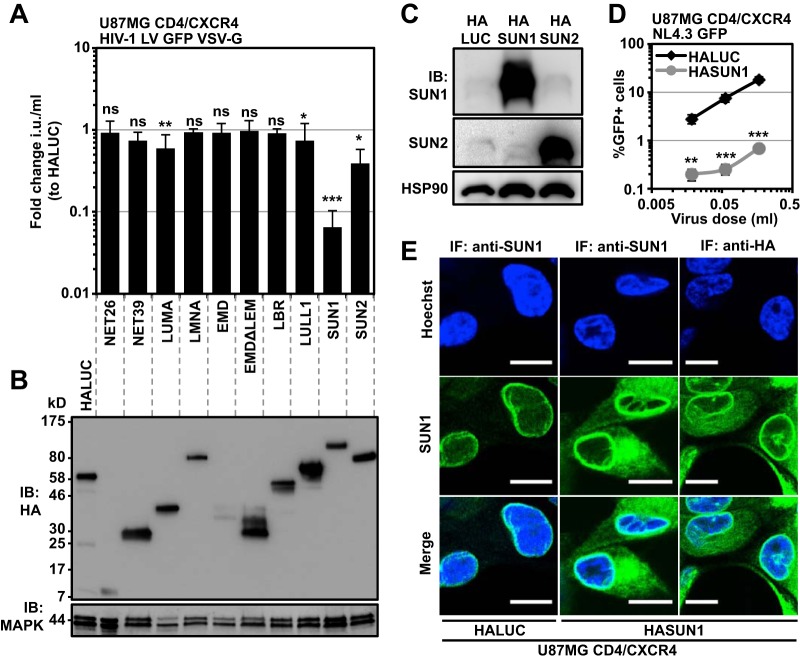
Overexpression of SUN1 or SUN2 inhibits HIV-1 infection. (A) Nontransduced U87MG CD4/CXCR4 cells (n.t.) or cells expressing the indicated HA-tagged membrane-associated NE proteins or luciferase (LUC; negative control) were infected with serial dilutions of VSV-G-pseudotyped HIV-1 GFP LV, and the infectious units (i.u.) per ml of inoculum were calculated. Fold changes in mean infectious titers relative to HALUC control cells and standard deviations were determined from at least three viral doses from four independent biological repeats. Unpaired two-tailed *t* tests were performed. ns, not statistically significant; *, *P* < 0.05; **, *P* < 0.01; ***, *P* < 0.001. (B) Samples parallel to those shown in panel A were used to determine protein expression levels by immunoblotting using an HA-specific antibody. MAPK served as the loading control. (C) U87MG CD4/CXCR4 cells expressing HALUC, HASUN1, or HASUN2 were probed with antibodies targeting SUN1 or SUN2. HSP90 served as a loading control. (D) U87MG CD4/CXCR4 cells expressing HALUC or HASUN1 were infected with NL4.3GFP reporter virus, and percentages of GFP-positive cells were determined 2 days later by flow cytometry. Multiple unpaired two-tailed *t* tests were performed. ***, *P* < 0.001; **, *P* < 0.0. (E) U87MG CD4/CXCR4 cells expressing HALUC or HASUN1 were subjected to immunofluorescence microscopy using HA- and SUN1-specific antibodies. Nuclei were visualized using Hoechst stain. Scale bar, 10 μm.

HIV-1 traverses the NE through NPCs and integrates preferentially in genomic areas that are topologically close to the INM (12). We aimed to identify membrane-associated NE proteins that impact the early stages of HIV-1 infection, and so we performed an overexpression screen with a series of hemagglutinin (HA)-tagged human NE proteins (Table 1). U87MG CD4/CXCR4 cells were stably transduced with lentiviral vectors (LVs) encoding the individual proteins, or with firefly luciferase (LUC) as a negative control, and were challenged with vesicular stomatitis virus G protein (VSV-G)-pseudotyped green fluorescent protein (GFP)-encoding HIV-1 LV. Infection was measured as the percentage of GFP-positive cells 2 days after challenge. While most proteins had minimal or no impact, overexpression of SUN2 resulted in an ∼5-fold reduction in HIV-1 LV infection (as described previously) (21, 53), while overexpression of SUN1 inhibited infection by ∼20-fold (Fig. 1A). We assessed protein expression levels by immunoblotting using an HA-specific antibody (Fig. 1B) and confirmed that SUN1 and SUN2 were well expressed. Indeed, the use of specific antibodies for SUN1 or SUN2 demonstrated that ectopically expressed proteins were substantially more abundant than endogenous SUN1/SUN2 (Fig. 1C). Importantly, the strong inhibition of HIV-1 infection by SUN1 was also observed when we used a full-length nonpseudotyped CXCR4-tropic HIV-1 GFP reporter virus (NL4.3GFP), indicating that the route of viral entry does not affect the magnitude of SUN1-mediated suppression and suggesting an effect downstream of membrane fusion (Fig. 1D). We also tested untagged SUN1 and observed a similarly strong block to HIV-1 infection (data not shown).

**TABLE 1:** Membrane-associated NE proteins analyzed in the overexpression screen

NE protein	Species	Tag^*a*^	Expression in WB assay (antibody)^*b*^	Cell viability^*c*^ (U87MG CD4/CXCR4)	Block of HIV-1 (U87MG CD4/CXCR4)
SUN1	Human	N-term HA	Yes (anti-HA)	++	20- to 50-fold
SUN1	Human	Untagged	Yes (anti-SUN1)	++	20- to 50-fold
SUN2	Human	N-term HA	Yes (anti-HA)	+	3- to 5-fold
SUN2	Human	Untagged	Yes (anti-SUN2)	+	3- to 5-fold
NET26	Human	N-term HA	Yes (anti-HA)	++	−
NET39	Human	N-term HA	Yes (anti-HA)	++	−
LUMA	Human	N-term HA	Yes (anti-HA)	+	2-fold
LMNA	Human	N-term HA	Yes (anti-HA)	++	−
EMD	Human	N-term HA	Yes (anti-HA)	+	−
EMDΔLEM	Human	N-term HA	Yes (anti-HA)	+	−
LBR	Human	N-term HA	Yes (anti-HA)	++	−
LULL1	Human	N-term HA	Yes (anti-HA)	++	2-fold
LULL1	Human	C-term HA	Yes (anti-HA)	+	−
TORSA	Human	N-term HA	No (anti-HA)	++	−
TORSA-EQ	Human	N-term HA	No (anti-HA)	++	−
TORSA	Human	C-term HA	Yes (anti-HA)	−	−
TORSA-EQ	Human	C-term HA	No (anti-HA)	−	−
TSPAN5	Human	C-term HA	No (anti-HA)	++	−
TMEM53	Human	C-term HA	Yes (anti-HA)	−	−
LAP2β	Human	C-term HA	Yes (anti-HA)	−	−
NURIM	Human	N-term HA	No (anti-HA)	++	−
NURIM	Human	C-term HA	Yes (anti-HA)	−	−
UNCL	Rat	N-term HA	Yes (anti-HA)	++	−
UNCL	Human	C-term HA	No (anti-HA)	++	−
NET3	Human	N-term HA	No (anti-HA)	++	−
NET3	Human	C-term HA	No (anti-HA)	+	−
NET31	Human	N-term HA	No (anti-HA)	++	−

a N-term HA, N-terminal HA; C-term HA, C-terminal HA.

b WB assay, Western blotting assay to detect protein expression with indicated antibody.

c Cell viability was visually judged by the appearance of cytopathic effect and cell death in culture, in comparison to parental U87MG CD4/CXCR4 cells (response set as 100%). ++, 90 to 100% relative to response in parental cells; +, 50% to 89%; −, 0 to 49%. Proteins for which no expression signal was observed or which showed a strong cytopathic effect were not investigated further.

When we compared the subcellular localization of endogenous SUN1 with ectopically expressed HASUN1 in U87MG cells, we found that the tagged protein retained a predominantly perinuclear staining pattern, but with some additional cytosolic localization (Fig. 1E). The SUN1 cDNA that was used in the experiments represents a recently identified splice isoform encoding 888 amino acids (see Material and Methods) (54). We also tested overexpression of the previously described SUN1 transcript variant 1, which encodes 785 amino acids (ENST00000401592.5), and found no phenotypic differences regarding the block to HIV-1 infection (data not shown).

### SUN1 overexpression inhibits HIV-1 and HIV-2, but not SIV_mac_, FIV, EIAV, or MoMLV.

**FIG 2:**
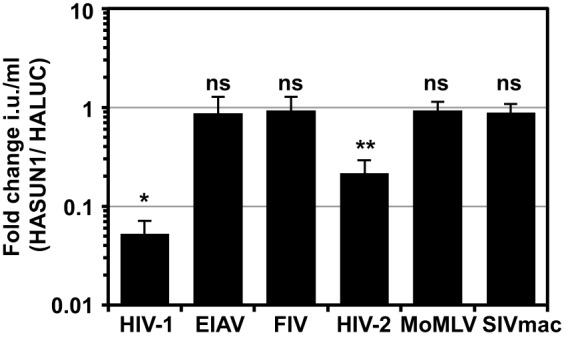
SUN1 inhibits HIV-1 and HIV-2_ROD_, but not SIV_mac_, FIV, EIAV, or MoMLV infection. U87MG CD4/CXCR4 cells expressing HALUC or HASUN1 were infected with serial dilutions of the indicated VSV-G-pseudotyped GFP retroviral vector, and infectious titers (infectious units [i.u.] per milliliter) were determined. Fold changes of infectious titers (HASUN1/HALUC) were calculated for at least eight different doses of two biological replicates for each vector, and mean values with standard deviations are shown. A paired two-tailed *t* test was performed. *, *P* < 0.05; **, *P* < 0.01; ns, not statistically significant.

We next investigated the selectivity of the SUN1-induced block for different retroviruses. U87MG CD4/CXCR4 cells expressing either HASUN1 or HALUC as negative controls were challenged with serial dilutions of a diverse set of VSV-G-pseudotyped GFP-encoding retroviral vectors derived from SIV_mac_, HIV-2_ROD_, feline immunodeficiency virus (FIV), equine infectious anemia virus (EIAV), or Moloney murine leukemia virus (MoMLV). SUN1 blocked HIV-1 GFP LV, as well as an LV derived from HIV-2_ROD_, whereas SIV_mac_, FIV, EIAV, and MoMLV were each unaffected by HASUN1 overexpression (Fig. 2). We observed a similar profile when we compared the infectious titers in cells overexpressing HASUN2 (data not shown). While HASUN1 overexpression inhibited HIV-1 infection by ∼20-fold, HIV-2_ROD_ was typically suppressed by ∼5-fold (Fig. 2), further indicating the virus substrate specificity.

### Overexpression of SUN1 reduces HIV-1 2-LTR circle accumulation.

**FIG 3:**
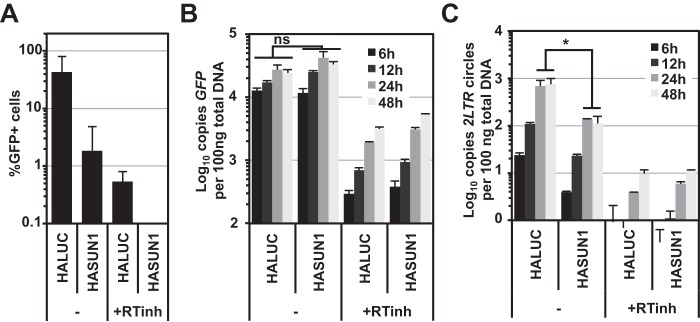
SUN1 inhibits HIV-1 2-LTR circle formation. (A) U87MG CD4/CXCR4 cells expressing HALUC or HASUN1 were infected in the presence or absence of 5 μM efavirenz (+RTinh) with HIV-1 GFP LV^++^. Total DNA was extracted at the indicated time points, and parallel samples were used to determine percentages of infected cells by flow cytometry 2 days postinfection. Mean infectivities for three samples and standard deviations are shown. (B and C) Early reverse transcription products for GFP (B) or 2-LTR circles (C) were measured by qPCR and normalized to total DNA input. Mean copy numbers per 100 ng total DNA for three independent samples with standard deviations are shown. Statistical analysis was performed using a paired two-tailed *t* test. ns, not statistically significant; *, *P* < 0.05.

To determine the stage of the infection block, we infected HALUC-expressing (negative control) or HASUN1-expressing U87MG CD4/CXCR4 cells with the VSV-G-pseudotyped HIV-1 GFP LV and isolated total DNA at various time points postinfection (Fig. 3). Quantitative TaqMan PCR was then used to determine the copy numbers of GFP or 2-long terminal repeat (2-LTR) circles, surrogates for early reverse transcription (RT) products and nuclear import, respectively. To control for plasmid contamination originating from vector production, we also included samples in which we added the allosteric reverse transcriptase inhibitor Efavirenz (+RTinh). Since LV-mediated transduction was used to express HASUN1 or HALUC, we used a reporter vector where the 5′- and 3′-LTRs had been engineered such that only the 2-LTR circles produced by the reporter virus could be detected (referred to as HIV-1 LV^++^). In a parallel sample, we measured infectivity by determining the percentage of GFP-expressing cells 48 h after infection. While cells overexpressing HASUN1 showed substantially reduced permissivity to HIV-1 LV^++^ infection compared to control cells, similar accumulations of the GFP cDNA product were observed in control and HASUN1-expressing cells, indicating that SUN1 overexpression did not suppress reverse transcription (Fig. 3A and B). In marked contrast, 2-LTR circle accumulation decreased by ∼10-fold in the presence of overexpressed SUN1, implying a block to nuclear entry or 2-LTR circle formation (Fig. 3C).

### Differential susceptibility of HIV-1 strains to SUN1-mediated inhibition.

**FIG 4:**
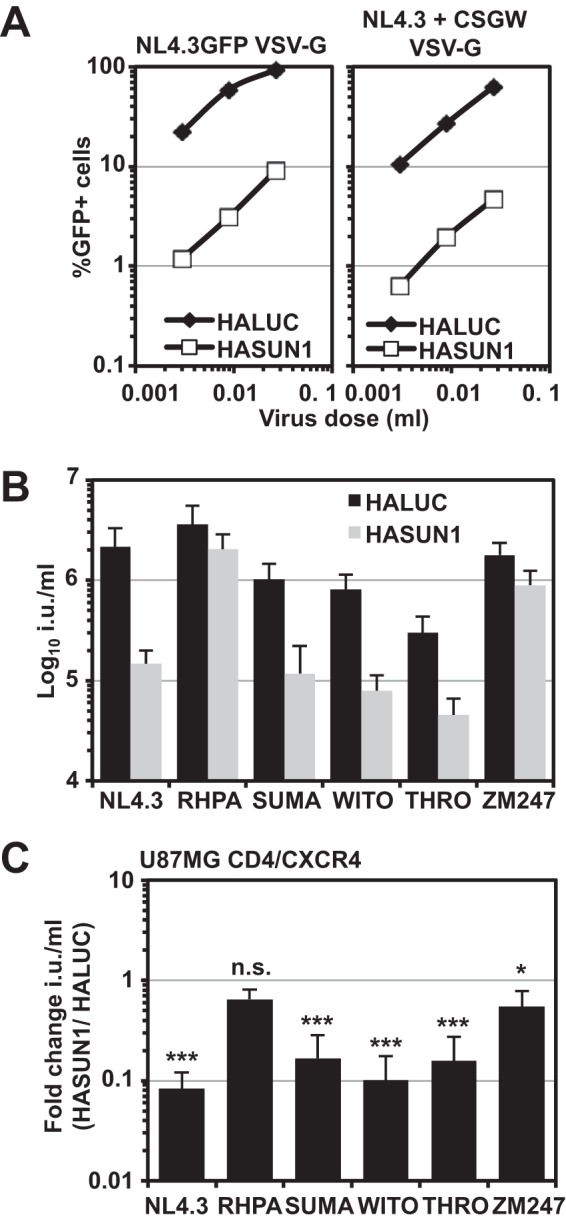
HIV-1 strains have differential sensitivities to the SUN1-induced block. (A) VSV-G-pseudotyped NL4.3GFP reporter virus or virus derived by cotransfecting NL4.3 with the GFP LV pCSGW was produced in parallel and titrated on U87MG CD4/CXCR4 cells expressing HALUC or HASUN1. Percentages of infected cells were determined 48 h later by flow cytometry. (B) U87MG CD4/CXCR4 cells expressing HALUC or HASUN1 were infected with the indicated VSV-G-pseudotyped virus produced by cotransfection with the HIV-1 GFP LV pCSGW, and infectious titers (in infectious units [i.u.] per milliliter) were determined from 10 viral doses of two independent biological replicates. (C) Presentation of the same data as shown in panel B, here with the fold changes (HASUN1/HALUC ratios) for mean titers with standard deviations. Statistical analysis was performed using an unpaired two-tailed *t* test. ns, not statistically significant; *, *P* < 0.05; ***, *P* < 0.001.

We next investigated whether the sensitivity toward SUN1-induced block varies among different HIV-1 strains. For rapid testing without the cumbersome derivation of reporter viruses, we applied a strategy of cotransfecting a GFP-encoding minigenome LV with different full-length HIV-1 molecular clones (55). First, we tested how this strategy compared with one using a typical GFP reporter virus. In parallel, we transfected 293T cells with a VSV-G-encoding plasmid and either NL4.3GFP or NL4.3 in combination with the GFP reporter vector pCSGW. We found that both virus preparations yielded similar infectious titers on 293T cells (data not shown). In addition, we analyzed inhibition by overexpressed SUN1 and found that in both cases infectivity was suppressed ∼20-fold by SUN1 (Fig. 4A). We then tested infection by the transmitted founder (T/F) viruses RHPA, SUMA, WITO, THRO, and ZM247 (Fig. 4B and C): SUMA, WITO, and THRO infectivities were all reduced by ∼6- to ∼10-fold by SUN1, whereas RHPA and ZM247 were largely unaffected (Fig. 4B and C). Similar reductions in sensitivities of these T/F viruses to SUN2-mediated inhibition suggested a common mechanism (data not shown). These data show that some HIV-1 strains are insensitive to SUN1- or SUN2-imposed barriers to infection.

### HIV-1 capsid protein determines sensitivity to SUN1-mediated inhibition.

**FIG 5:**
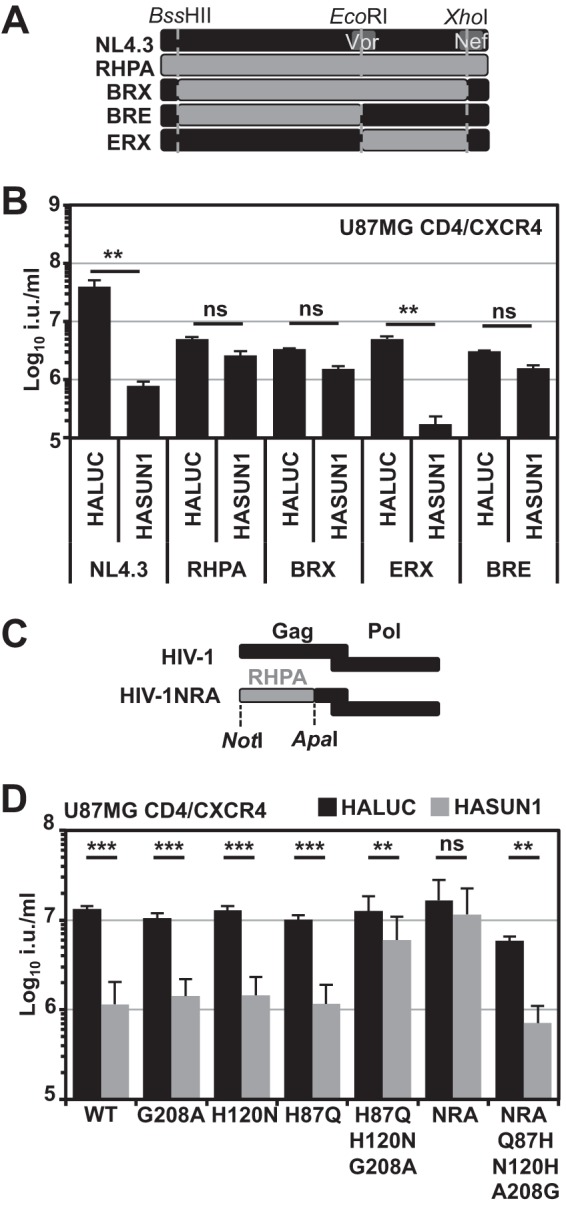
The determinant for the sensitivity to SUN1-induced inhibition maps to CA. (A) Schematic of the generation of chimeric viruses between T/F virus RHPA and NL4.3. (B) VSV-G-pseudotyped GFP reporter viruses were produced by cotransfection with the pCSGW vector, and U87MG CD4/CXCR4 cells were infected with serial dilutions. Infectious titers were determined from at least three different viral doses, and mean titers with standard deviations are shown. Representative results of three independent experiments are shown. Statistical analysis was performed using an unpaired two-tailed *t* test. ns, not statistically significant; **, *P* < 0.01. (C) Schematic of the generation of the HIV-1NRA Gag-Pol expression vector. (D) U87MG CD4/CXCR4 cells expressing HALUC or HASUN1 were infected with VSV-G-pseudotyped HIV-1 wild-type or NRA CA mutant GFP LVs, and mean infectious titers (in infectious units [i.u.] per milliliter) were determined from 10 different viral doses of three independent biological replicates. Fold changes in titers and standard deviations are shown. Statistical analysis was performed using an unpaired two-tailed *t* test. ns, not statistically significant; **, *P* < 0.01; ***, *P* < 0.001.

To identify the viral determinant(s) dictating sensitivity to SUN1 inhibition, we generated chimeric proviruses between the sensitive NL4.3 and the insensitive RHPA by using restriction sites BssHII (5′-LTR), EcoRI (Vpr), and XhoI (Nef), common to both proviruses (Fig. 5A). We produced GFP-reporter virus constructs by cotransfecting a GFP-encoding vector as described above and tested infectivity in U87MG CD/CXCR4 cells expressing HALUC or HASUN1 (Fig. 5B). Replacement of the sequence between the BssHII and EcoRI sites (encoding Gag, Pol, Vif, and part of Vpr) of NL4.3 with that of RHPA rendered the resulting virus (BRE) largely insensitive to SUN1 inhibition, while replacement of the EcoRI-XhoI fragment (encoding part of Vpr, Vpu, Env, Nef, Tat, and Rev) generated a chimeric virus (ERX) that was still sensitive to SUN1-mediated inhibition (Fig. 5B). We also observed that HIV-1 containing the CA-p2 of SIV_mac_ (3), like SIV_mac_, was largely insensitive to SUN1 or SUN2 (data not shown). We therefore inferred that the insensitivity of T/F viruses to SUN1 or SUN2 may be dictated by CA.

To further delineate the specific sequence determinant, we generated the Gag-Pol-encoding vector pCMV-ΔR8.91ExNRA (HIV-1NRA LV), which contained the RHPA Gag sequence up to the ApaI site (Fig. 5C). While wild-type HIV-1 LV (NL4.3-based) infectivity was reduced at least 10-fold by SUN1, HIV-1NRA LV was largely resistant (Fig. 5D). When we compared the RHPA and NL4.3 CA sequences, we found differences at nine positions, some of which were located proximal to, or within, the cyclophilin-binding loop. We therefore introduced single amino acid substitutions into NL4.3 CA to identify residues that would render the virus insensitive to SUN1-mediated inhibition. We found that all single amino acid substitutions that did not affect infectivity relative to the wild type also maintained sensitivity to SUN1 (Fig. 5D and data not shown).

We next engineered some compound mutants and found that the triple mutant H87Q+H120N+G208A rendered HIV-1 largely insensitive to SUN1-mediated inhibition, phenocopying HIV-1NRA LV (Fig. 5D). Reciprocally, a triple mutant with the reverse amino acid substitutions (Q87H+N120H+A208G) was introduced into HIV-1NRA and was found to be sufficient to render HIV-1NRA SUN1 sensitive (Fig. 5D). These data indicate that specific residues in HIV-1 CA are involved in conferring sensitivity to SUN1-induced inhibition.

### *In vitro*-assembled HIV-1 CANC complexes interact with SUN1 and SUN2.

**FIG 6:**
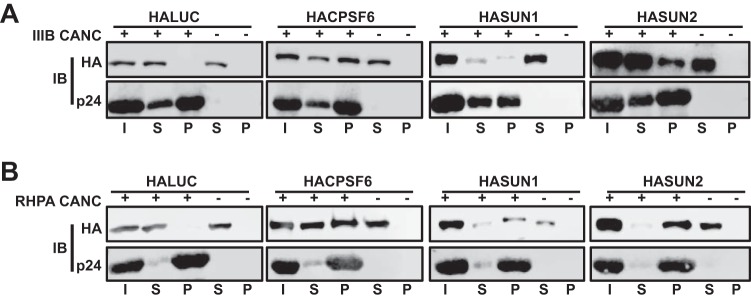
*In vitro*-synthesized HIV-1 CANC nanotubes capture SUN1 and SUN2 from cell lysates. (A) Cell lysates from 293T cells transfected with HALUC, HACPSF6, HASUN1, or HASUN2 were incubated with *in vitro*-synthesized CANC nanotubes before centrifugation through a sucrose cushion and analysis of supernatants and pellets by immunoblotting using HA- and CA-specific antibodies. I, input; S, supernatant; P, pellet. (B) *In vitro*-synthesized CANC complexes from RHPA were mixed with cell lysates and analyzed as described for the results shown in panel A.

Since HIV-1 sensitivity to SUN1- or SUN2-mediated suppression is determined by specific amino acid residues in CA (Fig. 5), we speculated that HIV-1 CA may interact with SUN1 or SUN2. To address this, we analyzed whether ectopically expressed HASUN1 or HASUN2 could be captured from 293T cell lysates when we used synthetic CANC nanotubes derived from HIV-1_IIIB_ or the T/F virus RHPA. As a positive control, we expressed HA-tagged CPSF6 (11) in 293T cells and found that CPSF6 interacted efficiently with IIIB and RHPA CANC nanotubes, while the negative control, HALUC, did not (Fig. 6A and B). Enrichment of proteins through binding to CANC complexes was controlled internally by our use of parallel samples without added CANC complexes. Both HASUN1 and HASUN2 interacted with IIIB CANC complexes (Fig. 6A); however, despite the insensitivity of RHPA infection to SUN1- or SUN2-mediated suppression, its corresponding CANC complexes also interacted with SUN1 and SUN2 in cell lysates (Fig. 6B). Thus, while SUN1 and SUN2 appear to interact with CANC complexes *in vitro*, we were unable to identify a straightforward correlation between such interactions and the suppression of virus infection in cultured cells (discussed below).

### The amino-terminal domain of SUN1 mediates HIV-1 inhibition.

**FIG 7:**
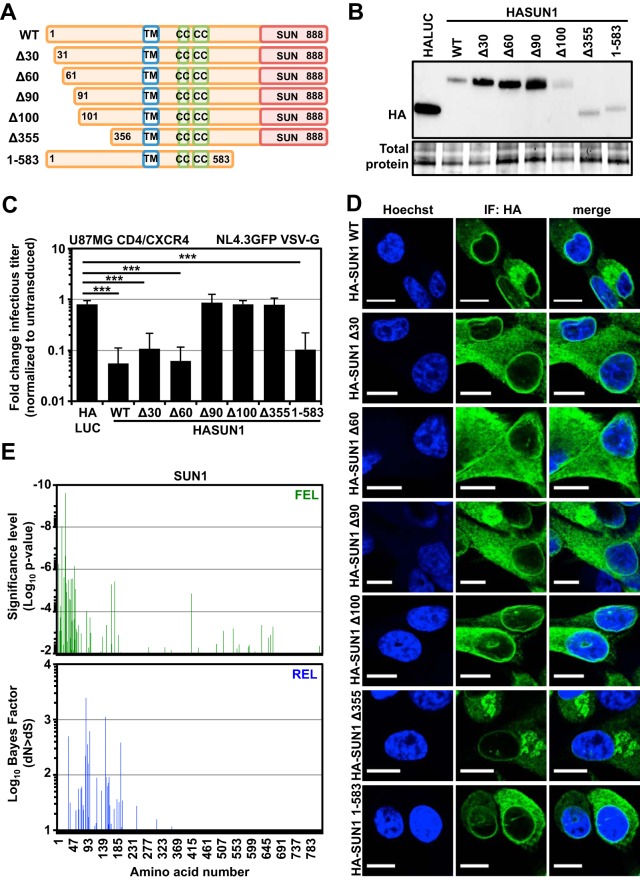
The amino-terminal domain of SUN1 is required for the block to HIV-1 and contains signatures of positive selection. (A) Schematic of analyzed SUN1 deletion mutants. (B) Immunoblot detection of HA-tagged proteins. Total protein measured by UV activation of the gel served as the loading control. (C) In parallel experiments to those for the samples shown in panel B, U87MG CD4/CXCR4 cells expressing HALUC, HASUN1, or the indicated SUN1 deletion mutants were infected with serial dilutions of VSV-G-pseudotyped NL4.3GFP, and infectious titers were determined. Mean fold changes (relative to untransduced control cells) of at least 10 infectious titers (in infectious units [i.u.] per milliliter) from three independent experiments and standard deviations are shown. Statistical analysis was performed using an unpaired two-tailed *t* test. ***, *P* < 0.001. (D) Immunofluorescence microscopy of U87MG CD4/CXCR4 cells expressing HASUN1 or HASUN1 deletion mutants stained with an HA-specific antibody. Nuclei were visualized using Hoechst stain. Scale bar, 10 μm. (E) Positive selection analysis using the FEL (top panel) and REL (bottom panel) methods.

To identify which regions of SUN1 are involved in the inhibition of HIV-1, we generated amino- and carboxy-terminal deletion mutants. When we analyzed amino-terminal SUN1 deletion mutants, we found that removal of the first 30 (HASUN1Δ30) or 60 (HASUN1Δ60) amino acids had no substantial effect on the ability of SUN1 to block HIV-1 (Fig. 7A, B, and C). However, deletion of 90 (HASUN1Δ90), 100 (HASUN1Δ100), or 355 (HASUN1Δ355) amino acids abrogated HIV-1 suppression (Fig. 7A and B and C). We also observed that SUN1 mutants lacking regions within the amino-terminal 90 amino acids had higher expression levels than the wild-type protein (Fig. 7B). Notably, the subcellular localizations of restricting wild-type HASUN1, HASUN1Δ30, or HASUN1Δ60 were similar to those of inactive HASUN1Δ90 or HASUN1Δ100, based on immunofluorescence microscopy, as they all displayed cytoplasmic and perinuclear staining (Fig. 7D). Deletion of the carboxy-terminal SUN domain (HASUN1 1-583) did not interfere with the inhibition of HIV-1 (Fig. 7C), suggesting that the interaction with nesprin proteins within the perinuclear space and LINC complex formation may not be required for the antiviral activity observed here.

Signatures of positively selected amino acid residues can be indicative of host-pathogen interactions (3, 56–59). The ratio of nonsynonymous to synonymous nucleotide changes at codon positions can be used to identify positively selected sites. We aligned SUN1 cDNA sequences from 23 species available from the OrthoMaM database and analyzed the alignment for positively selected sites by using the Datamonkey website and the FEL and REL substitution models (60, 61). We were able to identify signatures of evolutionary pressure within the amino-terminal region of SUN1 that were suggestive of possible interactions with pathogens (Fig. 7E).

**FIG 8:**
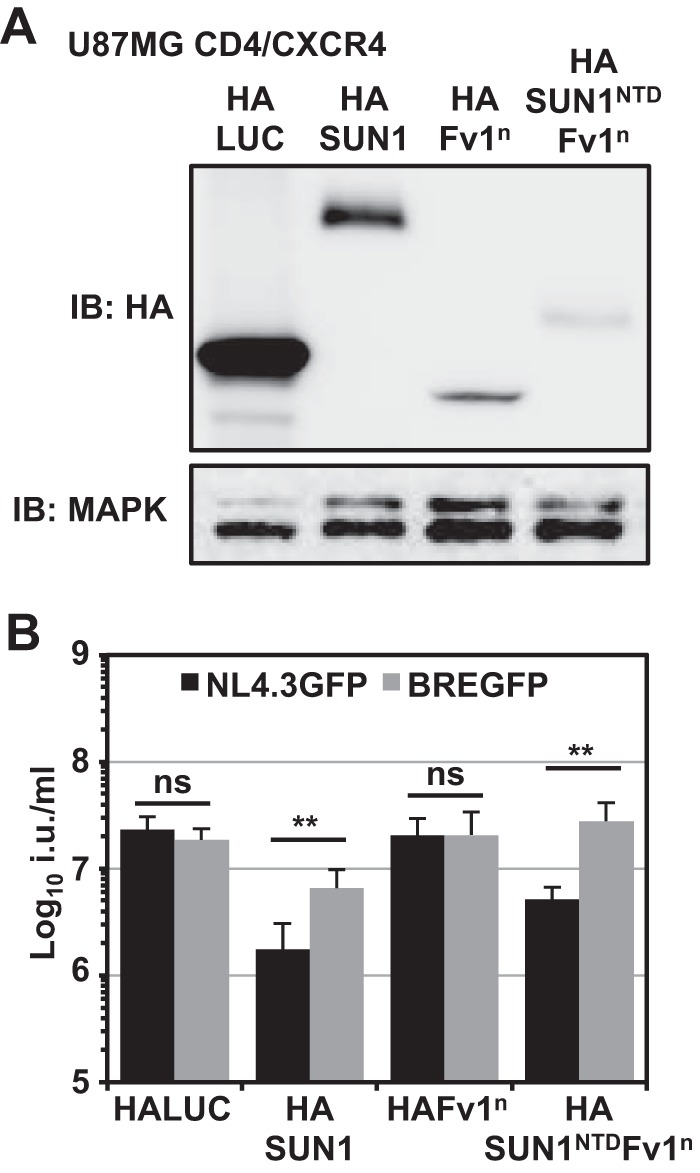
Fusion of the amino-terminal SUN1 domain to Fv1 generates a potent HIV-1-inhibiting factor. (A) Cell lysates of U87MG CD4/CXCR4 cells expressing HALUC (negative control), HASUN1 (positive control), HAFv1^n^, or a fusion protein of the amino-terminal 130 amino acids of SUN1 with Fv1^n^ were subjected to immunoblotting using an HA-specific antibody; MAPK served as the loading control. (B) Parallel cells (to those for which results are shown in panel A) were infected with serial dilutions of VSV-G-pseudotyped NL4.3GFP or BREGFP reporter viruses, and infectious titers were determined. Mean infectious titers (in infecious units [i.u.] per milliliter) with standard deviations were calculated from six independent viral doses of two biological replicates. Statistical analysis was performed using an unpaired two-tailed *t* test. ns, not statistically significant; **, *P* < 0.01.

We investigated whether the amino terminus of SUN1 is sufficient to inhibit HIV-1 infection by generating a fusion protein with the MLV restriction factor Friend virus susceptibility factor 1 (Fv1^n^). This strategy has been used to analyze CA binding of certain protein domains by creating Fv1-Cyp (62, 63) or MX2^NTD^-Fv1 (64) chimeras. As expected, expression of HAFv1^n^ in U87MG CD4/CXCR4 cells did not inhibit HIV-1 infection (Fig. 8A and B). In contrast, fusing the first 130 amino acids of SUN1 (N-terminal domain [NTD]) to the amino terminus of Fv1^n^ (HASUN1^NTD^-Fv1^n^) was sufficient to reduce HIV-1 infection (Fig. 8B), though the extent of inhibition did not reach the levels observed with HASUN1, most likely due to lower expression levels (Fig. 8A). In conclusion, the data suggest that, similar to SUN2 (21) (data not shown), the amino terminus of SUN1 is important for the inhibition of HIV-1 infection.

### CRISPR/Cas9-mediated *SUN2* but not *SUN1* gene disruption decreases HIV-1 infectivity.

**FIG 9:**
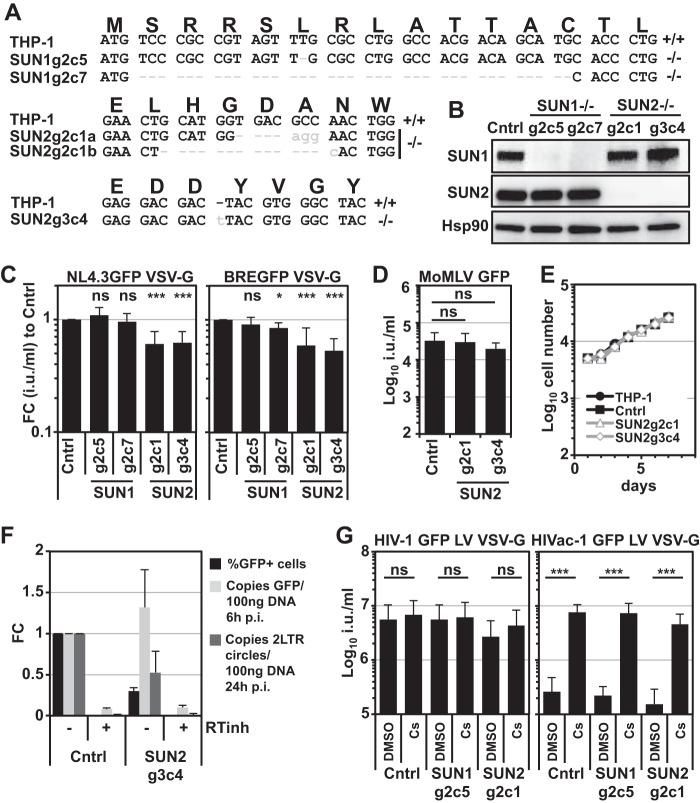
Gene disruption of *SUN2* but not *SUN1* reduces infectivity of HIV-1 in THP-1 cells. (A) THP-1 CRISPR/Cas9 single-cell clones transduced to express specific guide RNAs against SUN1 or SUN2 were generated. Single-cell cloning followed by PCR-based sequencing across the guide RNA target sites identified two clones for each gene in which the open reading frame was disrupted. (B) Disruption of gene expression was verified by immunoblotting using SUN1- or SUN2-specific antibodies; Hsp90 served as the loading control. CRISPR/Cas9 control cells (Cntrl) expressed an unrelated guide RNA. (C) SUN1- or SUN2-depleted cells were infected with serial dilutions of VSV-G-pseudotyped NL4.3GFP or BREGFP reporter viruses, and infectious titers were determined. Shown are the fold changes (FC) in mean titers compared to control cells determined from at least 10 viral doses of four independent experiments, with standard deviations. Statistical analysis was performed using a paired two-tailed *t* test. ns, not statistically significant; *, *P* < 0.05; ***, *P* < 0.001. (D) THP-1 control or SUN2 knockout cells were infected with VSV-G-pseudotyped MoMLV GFP LV. Average infectious titers were determined from a total of nine viral doses for three biological replicates. Error bars are standard deviations. Statistical analysis was performed using a nonpaired two-tailed *t* test. (E) Growth curves of SUN2-depleted THP-1 cells in comparison to THP-1 parental or control cells. (F) THP-1 control cells or SUN2 g3c4 knockout cells were infected with DNase-treated VSV-G-pseudotyped NL4.3GFP in the presence or absence of efavirenz (RTinh), and total DNA was isolated for TaqMan qPCR 6 h (GFP) or 24 h (2-LTR circles) postinfection (p.i.). Fold changes to control cells in the absence of RTinh were calculated. Data are means from two independent experiments, calculated from at least four replicates. Error bars are standard deviations. (G) THP-1 control cells, as well as SUN1 or SUN2 knockout cell lines, were infected with VSV-G-pseudotyped HIV-1 or HIVac-1 GFP LV in the presence of 5 μM CS or DMSO vehicle control. Shown are mean infectious titers (in infectious units [i.u.] per milliliter) with standard deviations from at least six virus doses of two independent experiments. Statistical analysis was performed using an unpaired two-tailed *t* test. ns, not statistically significant; ***, *P* < 0.001.

We next sought to address the role of endogenous SUN1 or SUN2 proteins in HIV-1 infection. We generated single-cell clones of THP-1 cells transduced with CRISPR/Cas9 LVs expressing individual specific guide RNAs (gRNAs). We generated two independent THP-1 single-cell knockout clones for each gene (SUN1g2-5, SUN1g2-7, SUN2g2-1, and SUN2g3-4). Gene disruption was validated by PCR sequencing across the gRNA target site as well as by immunoblotting (Fig. 9A and B). All lines were tested for their susceptibility to infection with VSV-G-pseudotyped NL4.3GFP or chimeric BREGFP reporter viruses. As shown in Fig. 9C, the depletion of SUN1 had no observable effect on infection by either virus, whereas the loss of SUN2 resulted in an ∼2- to 3-fold reduction in HIV-1 infection compared to a CRISPR/Cas9 control cell clone expressing an irrelevant control gRNA (Fig. 9C). We observed similar infection phenotypes in THP-1 cells that had been differentiated by use of phorbol 12-myristate 13-acetate (PMA) (data not shown). The infectivity defect in these SUN2 knockout cell clones could not be explained by defects in cell proliferation, since the infectious titer of a VSV-G-pseudotyped MoMLV GFP LV was unchanged in SUN2 knockout cells compared to control cells (Fig. 9D). We also determined growth kinetics for these cells and did not observe differences between parental THP-1, control, or SUN2 knockout lines (Fig. 9E). To identify the step in HIV-1 infection that was impaired in THP-1 cells lacking SUN2 expression, we infected control cells and SUN2 knockout g3c4 cells with DNase-treated VSV-G-pseudotyped NL4.3GFP and isolated total DNA at 6 or 24 h after infection for use in a TaqMan quantiative PCR (qPCR). In parallel, we measured infection 2 days postinfection by flow cytometry. We found that *GFP* reverse transcription products were present at similar levels in control and SUN2 knockout cells 6 h after infection (Fig. 9G). In contrast, levels of 2-LTR circles were reduced by ∼2-fold, which correlated with the decrease in infectivity (Fig. 9G), suggesting that reduced nuclear import underlies the decreased HIV-1 infectivity. These data support the idea that SUN2 helps promote the early stages of HIV-1 infection but that the contributions of SUN1 are less obvious, in that gene disruption does not yield an infection phenotype whereas purposeful overexpression can be a potent suppressor of infection. It is possible that SUN1 and SUN2 provide redundant functions for HIV-1 infection; however, simultaneous depletion of both proteins can result in mitotic defects and delayed cell proliferation (38), thus confounding the interpretation of virus infection experiments.

Lahaye et al. reported that in bone marrow-derived dendritic cells from mice, the infectivity of HIVac-1, an HIV-1 CA mutant that is restricted by CypA (65), is rescued from this restriction when SUN2 is absent (53). We tested whether infectivity of HIVac-1 could be rescued in THP-1 cells lacking SUN1 or SUN2. We observed no changes for wild-type HIV-1 GFP LV in the presence or absence of cyclosporine (CS) (Fig. 9G). The infectivity of HIVac-1 GFP LV was severely reduced in THP-1 control cells as well as in THP-1 SUN1 or SUN2 knockout cell clones, and CS treatment rescued infectivity in all cases to infection levels of the wild-type LV (Fig. 9G). We obtained similar results in Jurkat T cell lines with a *SUN1* or *SUN2* gene knockout (data not shown). The results suggest that the CypA effects on HIV-1 infection are not changed by *SUN1* or *SUN2* gene knockout.

## DISCUSSION

We have investigated the impact of several NE proteins on early HIV-1 infection by using a focused overexpression screen. We found that ectopic expression of UNC-84A/SUN1 or UNC-84B/SUN2 inhibits HIV-1 infection (Fig. 1). We concentrated our further studies on the effects of SUN1, since the magnitude of the block was substantially stronger than that for SUN2 (Fig. 1). We found that ectopically expressed SUN1 localized to the NE as well as to cytoplasmic regions (Fig. 1E). As a component of the LINC complex, the amino terminus of SUN1 interacts with proteins of the nuclear lamina, including lamin A/C, while the carboxy terminus interacts in the perinuclear space with the nesprin KASH domain (25, 66). Both SUN1 and SUN2 have also been reported to interact with the INM protein EMD (24). A previous study suggested that EMD serves as a cofactor for HIV-1 integration (67); however, later studies did not confirm this (68, 69), and the inability of a LEM domain-deleted EMD to reduce HIV-1 infection can be considered consistent with the latter reports (Fig. 1).

SUN2 had previously emerged from a functional cDNA screen as a type I interferon (IFN)-induced gene that blocks HIV-1 infection (22). During the preparation of the manuscript, three further studies were published reporting a possible role for SUN2 in early HIV-1 infection steps (21, 53, 70). In agreement with these studies, we did not observe changes in SUN2 protein levels in type I IFN-treated primary monocyte-derived macrophages, CD4^+^ T cells, or THP-1 cells (21, 53) (data not shown). Similar to what was observed by Donahue et al. for SUN2, we found that SUN1 overexpression also reduced HIV-1 2-LTR circle formation, a surrogate for nuclear import (Fig. 3). However, we did not find that SUN1 expression induced alterations in the morphology of nuclei, as has been observed for SUN2 (21). We also noted that overexpression of SUN1 was substantially less cytotoxic than SUN2 (data not shown), indicating differential effects on general cell function.

In agreement with our results, Donahue et al. showed that SUN2 expression caused a block to HIV-1_NL4.3_ and HIV-2_ROD_, while other strains, in particular many T/F viruses, were resistant and that the CA protein was likely the viral target (21). We observed a similar, albeit less broadly distributed, reduced sensitivity of some T/F viruses (i.e., RHPA and ZM247) to SUN1-mediated inhibition (Fig. 4). Notably, some T/F viruses, including RHPA, are hyposensitive to overexpressed MX2, which could indicate that these viruses generally bypass different cellular factors that are associated with the NE (71). The basis for the insensitivity of SIV_mac_ and a series of nonprimate retroviruses to human SUN1 and SUN2 overexpression (Fig. 2) is unknown at present, though one possibility is that these viruses may specifically interact with the SUN proteins of their natural hosts. Our results suggest that the amino terminus of SUN1 may have undergone processes of positive selection during evolution, which could indicate historical instances of species-specific viral interactions (Fig. 7E). Finally, it is worth recognizing that despite the multiple possible redundant cellular functions of SUN1 and SUN2, both proteins may also have individual roles; for example, while SUN2 has been described as a Rab5-binding protein involved in endosomal trafficking (72), no such function has been described for SUN1.

While Donahue et al. propagated an HIV-1 CA mutant (P207S) that was able to overcome the block induced by SUN2 overexpression, this residue is conserved between NL4.3 and the T/F virus RHPA and therefore cannot fully explain the insensitivity of RHPA. Our data suggested that the viral determinant for SUN1- as well as SUN2-mediated inhibition of HIV-1 infection is located in the HIV-1 CA protein; for example, HIV-1 harboring the CA from SIV_mac_ was largely insensitive to SUN1 (data not shown). Accordingly, we concentrated on differences between the RHPA and NL4.3 CA sequences and found that amino acid substitutions at positions 87, 120, and 208 in NL4.3 rendered the virus largely insensitive to SUN1 (Fig. 5) or SUN2 (data not shown). Intriguingly, HIV-2 was also sensitive to SUN1- and SUN2-induced inhibition of infection (Fig. 2 and data not shown). HIV-1 NL4.3 and HIV-2_ROD9_ CA amino acid sequences share only 69% identity, and amino acid residues corresponding to H/Q87, H/N120, and G/A208 in HIV-1 CA are P86, R118, and N207 in HIV-2_ROD9_ CA. Whether these residues are critical for the sensitivity of HIV-2 to SUN1- or SUN2-induced inhibition will require further investigation, especially given that these residues are conserved between HIV-2_ROD9_ and SIV_mac_, a virus that is insensitive to the SUN1- or SUN2-mediated block (Fig. 2).

These findings led us to test for interactions between SUN1 and CA, using *in vitro*-assembled CANC nanotubes. We found that SUN1 as well as SUN2 could be enriched from cell lysates when we used CANC complexes derived from a susceptible virus strain (Fig. 6). Surprisingly, however, RHPA CANC complexes also precipitated SUN1 and SUN2, suggesting that the insensitivity of RHPA to SUN1/2 may not simply be caused by a defect in binding. Similarly, insensitivity to MX2 restriction of certain CA mutants could not be correlated with defects in MX2 binding *in vitro* (73). SUN1 interacts with NPCs (47), potentially via (i) the NPC component NUP153 (74), a protein that is important for HIV-1 nuclear import (2, 11) and whose depletion causes changes in SUN1 localization (75), and/or (ii) the NUP153-associated protein POM121 (76), suggesting close proximity between SUN1 and NPCs. It is therefore possible that SUN1 overexpression and (experimentally forced) cytoplasmic accumulation inhibit HIV-1 infection by perturbing the orderly interaction of incoming HIV-1 (CA-containing) replication complexes with NPC components, or through indirect and unanticipated effects on NPC function. However, we think it is unlikely that SUN1 overexpression simply interferes with NUP153 activity, since SIV_mac_ is insensitive to SUN1- or SUN2-induced inhibition (Fig. 2) but is equally sensitive to RNA interference-mediated NUP153 depletion as are HIV-2 and HIV-1 (2).

We investigated which domains of SUN1 are important for the inhibition of HIV-1 and found that deletion of the carboxy terminus did not impact the block (Fig. 7). This suggests that interaction of SUN1 with nesprins in the ONM is not important for HIV-1 inhibition (25). In contrast, deleting 90 amino acids from the amino terminus completely disrupted the antiviral activity (Fig. 7). Not only was the amino terminus required for antiviral activity, but it was also sufficient when artificially fused to Fv1^n^, generating a potent HIV-1-inhibiting factor (Fig. 8). Since this chimeric SUN1^NTD^-Fv1^n^ localizes to the cytoplasm (data not shown), we inferred that the interaction of the SUN1 amino-terminal domain with incoming HIV-1 CA can occur in the cytoplasm.

We addressed the contributions of the endogenous SUN1 and SUN2 proteins to HIV-1 infection in the myeloid cell line THP-1 (Fig. 9) by generating single-cell clones in which *SUN1* or *SUN2* had been knocked out. We found a moderate but reproducible decrease in HIV-1 infection (but not with MoMLV) in the absence of SUN2, in agreement with published data using shRNA-mediated SUN2 reduction (65), while there was no effect on HIV-1 infection in *SUN1* CRISPR/Cas9 cells (Fig. 9). The same pattern was also seen for the chimeric virus BRE, which contains CA from the T/F virus RHPA and has reduced sensitivity to the block that results from induction of SUN1 or SUN2 overexpression. Despite a lack of sensitivity to ectopically expressed SUN2, it is possible that RHPA CA may still be able to interact with SUN2, since an *in vitro* interaction between CANC complexes and SUN2 can occur (Fig. 6). In contrast to other researchers (53), we found that the lack of SUN2 in THP-1 cells reduced 2-LTR circle accumulation, indicative of a defect in virus nuclear import (Fig. 9). A series of central questions therefore arises from this work, including addressing the basis for assay-dependent differences between viral strains and understanding why infection by viruses such as NL4.3 is reduced by either SUN2 overexpression or endogenous gene knockout.

Lahaye et al. have proposed that SUN2 promotes CypA-dependent steps during HIV-1 infection (53). This conclusion was based on two major observations. First, bone marrow-derived dendritic cells from *SUN2*^−/−^ mice display increased permissivity to infection by an HIV-1 mutant that is hypersensitive to blockade by CypA (referred to as HIVac-1) (65). Second, shRNA-mediated depletion of SUN2 in human primary CD4^+^ T cells decreased permissivity to HIV-1 infection and the inhibition of CypA function only had a minor effect in SUN2-depleted cells, while in control cells CypA inhibition caused a significantly greater reduction in HIV-1 infection (65). We challenged this conclusion by infecting THP-1 *SUN1* or *SUN2* knockout cells with either wild-type HIV-1 GFP LV or HIVac-1 in the presence or absence of the cyclophilin inhibitor CS. In all cases, irrespective of SUN1/2 status, the infectivity of HIVac-1 was substantially reduced in the absence of CS, and addition of CS rescued infectivity to levels comparable to those of the wild type (Fig. 9). In agreement with our results, a recent study found no connection between SUN2 and CypA, even in primary CD4^+^ T cells (70). While there may be differences between primary CD4^+^ T cells and immortalized cell lines, we conclude that gene disruption of *SUN1* or *SUN2* in the studied cell lines has no impact on the CypA-mediated events during early HIV-1 postentry steps. To address the important question of functional redundancy between *SUN1* and *SUN2*, we attempted simultaneous depletion of both genes; however, severe effects on cell proliferation were evident (38), and we were unable to generate double-knockout cell lines. Therefore, we have not yet been able to determine whether SUN1 and SUN2 have redundant activities in the context of HIV-1 postentry steps.

In conclusion, we propose that the amino-terminal domains of SUN1 and SUN2 may interact with incoming HIV-1 subviral CA-containing replication complexes and facilitate steps during early HIV-1 infection, possibly in a redundant fashion. The CA protein has been demonstrated to be important for HIV-1 nuclear import (3, 11, 77, 78), and we showed here that the amino-terminal domain of SUN1 interacts with HIV-1 in a CA-specific manner. Because the endogenous SUN1 and SUN2 proteins localize to the INM and their amino termini face the nucleoplasm, we hypothesize that their interactions with nuclear CA-containing nucleoprotein complexes (79–81) may contribute to nuclear import and/or targeting of the HIV-1 integration machinery to the host cell genome.

## MATERIALS AND METHODS

### Cells.

THP-1 cells were grown in RPMI 1640 GlutaMax medium (Gibco) supplemented with 10% heat-inactivated fetal calf serum (FCS), 100 U/ml penicillin, and 100 μg/ml streptomycin. For differentiation, THP-1 cells were treated with 25 ng/ml PMA (Sigma-Aldrich) overnight. 293T and U87MG CD4/CXCR4 cells (82) were grown in Dulbecco's modified Eagle medium (DMEM GlutaMax; Gibco) with 10% heat-inactivated FCS and penicillin-streptomycin.

### Plasmids, cDNAs, and viral vectors.

cDNAs for EMD and EMDΔLEM were kind gifts of Juan Martin-Serrano (King's College London). SUN1, SUN2, and LMNA cDNAs were derived from THP-1 total RNA using the SuperScript III One-Step RT-PCR system with Platinum *Taq* DNA polymerase (ThermoFisher Scientific) cloned into TopoTA pCR2.1 (ThermoFisher Scientific) and subcloned into pCSxW (83). The SUN1 isoform cloned and used in the assays encoded 888 amino acids and represents a novel isoform with closest similarity to isoform ENST00000405266 but containing an additional exon (ENSE00003501736), which has been described in other alternatively spliced SUN1 isoforms (e.g., ENST00000429178.5) and with a described common single nucleotide polymorphism (SNP; rs6461378), leading to the H118Y amino acid change. The LULL1, LBR, NET26, NET39, and LUMA cDNAs were a gift from Rose Goodchild (KU Leuven) and Barbara Klupp (84) and were cloned into pCSxW following PCR amplification. HAFv1^n^ cDNA was amplified from pEXN-Fv1^n^ (62) by PCR and inserted into pCSxW. To generate HASUN1^NTD^-Fv1^n^ the fragment encoding the SUN1 amino terminus (amino acids 1 to 130) was subcloned into pCSxW-HAFv1^n^. For overexpression studies, proteins were usually expressed as amino-terminal HA-tagged fusion proteins from pCSxW-HA (83).

VSV-G pseudotyped GFP-encoding HIV-1 vectors were produced using pCMV-ΔR8.91 or the derivative thereof, pCMV-ΔR8.91Ex-NRA. pCSGW, and pMD.G have been described previously (3). pCMV-ΔR8.91Ex containing a NotI site upstream of Gag and a unique ApaI site in Gag was a kind gift by Yasuhiro Ikeda (Mayo Clinic, Rochester, MN, USA). To generate pCMV-ΔR8.91Ex-NRA, the RHPA Gag sequence was amplified using forward primer 5′-AT CGC GGC CGC TGG TGA GAG *ATG* GGT GCG AGA GCG TCG GTA TTA AGC GGG GG-3′ (underlining in the primer sequence indicates the NotI restriction site, and italics indicate the start codon) and a reverse primer downstream of the ApaI site in RHPA and inserted between NotI/ApaI sites in pCMV-ΔR8.91Ex.

pCSGW^++^ was generated by site-directed mutagenesis, using 3′-LTR forward/reverse primers 5′-ACT GGA AGG GCT TTA AGA CTC CCA ACG AAG AC-3′/5′-TTC GTT GGG AGT CTT AAA GCC CTT CCA GTC CC-3′ and 5′-LTR primers 5′-ATC CCT CAG ACC GAA AAA GTC AGT GTG GAA AAT C-3′/5′-TCC ACA CTG ACT TTT TCG GTC TGA GGG ATC TCT AG-3′. To generate a 2-LTR standard plasmid, a PCR amplicon using forward/reverse primers 5′-AAC TAG AGA TCC CTC AGA CCC TTT T-3′/5′-CTT GTC TTC GTT GGG AGT GAA TT-3′ was generated from total DNA of cells transduced with pCSGW-derived vector and inserted into pCR2.1-TOPO. The generated plasmid was used to make a standard specific for pCSGW^++^ by site-directed mutagenesis.

Full-length HIV-1 GFP reporter virus preparations were generated from pNLENG-IRES-Nef (3, 85). Plasmids encoding NL4.3/RHPA chimeric viruses were generated by subcloning BssHII-, EcoRI-, and XhoI-digested RHPA plasmid fragments into pNL4.3. NL4.3 encoding Renilla luciferase in place of Nef has been described before (82). Plasmids to generate viral GFP reporter vectors of SIV_MAC_, HIV-2_ROD_, FIV, EIAV, and MoMLV have been described before (86).

To generate CRISPR/Cas9 cells, gRNA encoding oligonucleotides (MWG/Eurofins) were annealed and cloned into BsmBI-linearized plentiCRISPRv2 according to reported guidelines (Addgene) (87, 88). Oligonucleotides (forward/reverse) for cloning plentiCRISPRv2-SUN1g1were 5′-CAC CGT ACG TGT AGC CCG TGT TCT C-3′/5′-AAA CGA GAA CAC GGG CTA CAC GTA C-3′; for SUN1g2, 5′-CAC CGT CGT GGC CAG GCG CAA ACT A-3′/5′-AAA CTA GTT TGC GCC TGG CCA CGA C-3′; for SUN2g1, 5′-CAC CGC GCC TCA CGC GCT ACT CCC A-3′/5′-AAA CTG GGA GTA GCG CGT GAG GCG C-3′; for SUN2g2, 5′-CAC CGA ACT GCA TGG TGA CGC CAA C-3′/5′-AAA CGT TGG CGT CAC CAT GCA GTT C-3′; for SUN2g3, 5′-CAC CGC TCC TCT GAG GAC GAC TAC G-3′/5′-AAA CCG TAG TCG TCC TCA GAG GAG C-3′.

### SIV_mac_ VLP, lentiviral vector, and HIV-1 production.

293 T cells grown in 10-cm plates were transfected at a confluence of ∼75% with 4.5 μg HIV-1 viral vector plasmid pCSxW, 3 μg pCMVΔR8.91, and 3 μg pMD.G, using 4 μg polyethylenimine (PEI) per μg DNA in 1 ml of Opti-MEM (Gibco) per plate. For SIV_mac_ virus-like particle (VLP) production, 5 plates were transfected with 8 μg Gag-Pol-encoding plasmid pSIV3+ (89) and 2 μg pMD.G per plate. For VSV-G-pseudotyped full-length HIV-1 production, 8 μg HIV-1 GFP reporter viral plasmid and 2 μg pMD.G were transfected per plate. To generate full-length laboratory strains HIV-1_NL4.3_, HIV-1_IIIB_, or HIV-1 transmitted/founder RHPA, SUMA, WITO, THRO or ZM247 with a copackaged GFP lentivirus (LV) minigenome, 4.5 μg pCSGW was cotransfected with 3 μg full-length viral plasmid and 3 μg pMD.G. For all viral production, medium was changed 24 h posttransfection, the viral supernatant was harvested at 48 h and 72 h posttransfection, and collections were pooled and passed through a 0.45-μm filter (Rotilabo KH55.1, Roth). Depending on the experiment, viral vector supernatants were subjected to sucrose cushion purification as described previously (90). For SIV_mac_ VLP production, 30 ml viral supernatant was sucrose concentrated and resuspended in 300 μl RPMI 1640 containing penicillin-streptomycin.

### Generation of CRISPR/Cas THP-1 cell clones.

THP-1 CRISPR/Cas9 cells were generated by transduction with VSV-G-pseudotyped HIV-1 LV produced using pCMV-ΔR8.91, pMD.G, and plentiCRISPRv2 (Addgene) (87, 88). Transduced cell populations were selected with 2 μg/ml puromycin (Sigma-Aldrich) for 2 weeks. Single-cell clones were generated by limiting dilution in 96-well plates and grown for at least 2 weeks in the absence of puromycin. Cell clones without protein expression were determined by immunoblotting using SUN1- or SUN2-specific antibodies. To verify gene disruption, we sequenced PCR amplicons over the gRNA target site generated from isolated total genomic DNA of selected clones. The following primer pairs (forward/reverse) were used: for SUN1g1, 5′-ACA CTG CTG CTG GCC GTG TTT CCT G-3′/5′-AGT CAC CAG GAT GAA CAG ATT CAG-3′; for SUN1g2, 5′-AGT AAT AGT TGC TCT TGA AAA TCC AC-3′/5′-TCG AGA CAG GGT GCG GCT TTA CAG AC-3′; for SUN2g1 5′-ACA GTG CAG GGG TGC TTC ACA GAT C-3′/5′-TGC TGT GTG CTC ATA CAC ATG GAG C-3′; for SUN2g2, 5′-TTG TAA AGT TTG AAT GTG GC-3′/5′-AAG TCC TCG GTG GCC TTG CG-3′; for SUN2g3 5′-TGT TGG CCT TAG GTT GCC ATA G-3′/5′-AGC ACC CAC CAT GTG TGA GC-3′. As negative controls, we used either a THP-1 single-cell clone that has been described before (91) or parental THP-1 cells.

### Infections.

U87MG CD4/CXCR4 or THP-1 cells were plated at 8 × 10^4^ or 1 × 10^5^ cells, respectively, in 96-well plates. Cells were infected for 24 to 72 h before harvest, fixed in PBS plus 4% paraformaldehyde (PFA), and analyzed by flow cytometry using a FACSVerse system (BD Biosciences). Infectivities were determined as the percentage of GFP^+^ cells. Virus titrations were usually performed with 3-fold serial dilutions of the viral supernatant. CS (Sandimmune, Novartis Pharmaceutical Corporation) was used at 5 μM in dimethyl sulfoxide (DMSO) and added at the time of infection with the reporter virus. DMSO was used as the vehicle control at the same concentration.

### Immunoblotting and antibodies.

Proteins were separated in SDS-polyacrylamide gels by using the Mini-Protean Tetra cell system (Bio-Rad) at 120 V for 1 h and transferred to nitrocellulose or polyvinylidene difluoride membranes. Antibodies used in immunoblotting were the following: rat anti-HA coupled to peroxidase-conjugated monoclonal antibody 3F10 (1:5,000; Roche), rabbit anti-mitogen-activated protein kinase (anti-MAPK; Erk1/2; 1:1,000; Cell Signaling), mouse anti-HIVp24 (24.2) (92), monoclonal rabbit anti-SUN1 GTX63537 (1:1,000; Genetex), monoclonal rabbit anti-SUN2 EPR6557 (1:1,000; Abcam), rabbit polyclonal anti-Hsp90 antibody H-114 (1:3,000; sc-7947; Santa Cruz Biotechnology), and mouse anti-alpha-tubulin (1:3,000; Sigma-Aldrich). Secondary antibodies were goat anti-mouse IgG horseradish peroxidase (HRP) linked (1:10,000; Pierce, Thermo Scientific) and goat anti-rabbit IgG HRP-linked (1:10,000; Cell Signaling, NEB). Total protein input was determined via UV activation of Mini-Protean TGX stain-free reagent (Bio-Rad).

### Protein expression and purification.

HIV-1 CANC DNA sequences (Gag residues 133 to 432) derived from wild-type HIV-1_IIIB_ or from the founder virus strain RHPA were cloned into the pST39 vector (93) by using NdeI and BamHI sites. Proteins were expressed in Escherichia coli Rosetta (DE3) cells (Merck Millipore) following the protocol described by Lemke et al. (94) with some modifications. Briefly, a 20-ml overnight preculture was added to 300 ml of fresh Luria broth and grown for 4 h at 37°C. Then, three fractions of 100 ml of this culture were added to 1 liter of fresh broth and grown at 37°C until the optical density at 600 nm was 0.6. Protein expression was induced with 1 mM isopropyl-β-d-thiogalactopyranoside (IPTG) treatment for 5 h at 30°C. Cells were collected by centrifugation at 3,500 rpm for 20 min and lysed by sonication in a 50 mM Tris-HCl (pH 8.3) buffer containing 1 M NaCl, 10% glycerol, 1 mM EDTA, 10 mM dithiothreitol (DTT), and 1 mM phenylmethylsulfonyl fluoride (PMSF). Cell debris was removed by centrifugation at 30,000 × *g* for 20 min, and the supernatant was diluted with lysis buffer without salt to a final concentration of 0.5 M NaCl. Nucleic acids were removed by adding 0.11 volumes of 2 M ammonium sulfate and the same volume of 10% PEI (pH 8.0), stirring the sample for 20 min at 4°C, and finally centrifuging at 30,000 × *g* for 20 min. CANC was precipitated from the resulting supernatant by adding 0.35 volumes of saturated ammonium sulfate followed by 10,000 × *g* centrifugation for 15 min. Pelleted protein was resuspended in a 50 mM morpholineethanesulfonic acid (MES; pH 6.5) buffer containing 0.5 M NaCl, 10% glycerol, 1 mM EDTA, 1 mM DTT, and 1 mM PMSF and then diluted with the same buffer without salt to a final concentration of 0.2 M NaCl. The sample was cleared by centrifugation, and CANC was purified by cation-exchange chromatography on a 5-ml HiTrap SP HP column (GE Healthcare) using a 50 mM MES (pH 6.5) buffer containing 0.2 M NaCl, 10% glycerol, 1 mM EDTA, 1 mM DTT, and 1 mM PMSF as equilibration buffer. CANC was eluted with a linear gradient resulting from mixing the equilibration buffer with a 50 mM MES (pH 6.5) buffer, containing 1 M NaCl, 10% glycerol, 1 mM EDTA, 1 mM DTT, and 1 mM PMSF. Fractions containing the protein were pooled, and CANC was precipitated by adding 1 volume of saturated ammonium sulfate. Finally, the protein was resuspended to a final concentration of 200 μM in a 30 mM MES (pH 6) buffer containing 0.5 M NaCl, 1 mM EDTA, and 10 mM DTT.

### *In vitro* assembly of CANC complexes.

Purified CANC was assembled by mixing the protein to a 40 μM final concentration with 5 μM TG50 oligonucleotide (5′-TGT GTG TGT GTG TGT GTG TGT GTG TGT GTG TGT GTG TGT GTG TGT GTG TG-3′) in a 50 mM Tris-HCl (pH 8) buffer containing 100 mM NaCl and incubated overnight at room temperature.

### CANC pulldown.

293T cells transfected with pCSxW expressing HA-tagged firefly luciferase, CPSF6, or wild-type SUN1 or SUN2 proteins were lysed with hypotonic lysis buffer (10 mM Tris-HCl [pH 8], 10 mM KCl, 1× protease inhibitor cocktail [Roche]) using a Dounce homogenizer. Lysates were cleared by centrifugation at 20,000 × *g* for 15 min. For pulldown experiments, 200 μl of cell lysate was mixed with either 40 μl of 40 μM assembled CANC (an input sample was taken from this mix) or 40 μl of CANC binding buffer (50 mM Tris-HCl [pH 8], 100 mM NaCl) containing 5 μM TG50 and incubated at room temperature for 1 h. The mixture was then overlaid onto a 250 μl 70% sucrose cushion and centrifuged at 15,000 × *g* for 10 min. A sample of the supernatant was withdrawn for further analysis, and the pellet was washed with 500 μl of wash buffer (50 mM Tris-HCl [pH 8], 50 mM NaCl, and 5 mM KCl), and centrifuged again at 10,000 × *g* for 5 min. Finally, the pellet was resuspended in 50 μl of 1× SDS-PAGE loading buffer. Input, supernatant, and pellet fractions were analyzed by immunoblotting using appropriate antibodies.

### TaqMan qPCR.

U87MG CD4/CXCR4 HA-LUC or HA-SUN1 were seeded at 2 × 10^5^ cells per well in 6-well plates and infected the next day in triplicate with VSV-G-pseudotyped HIV-1 GFP LV containing the pCSGW^++^ minigenome in the presence or absence of the reverse transcription inhibitor efavirenz (5 μM; brand name Sustiva, 30-mg/ml oral solution; Bristol Meyer Squibbs) to control for plasmid contamination. THP-1 CRISPR/Cas9 control or SUN2 knockout g3c4 cells were seeded at 6 × 10^5^ cells per well in 6-well plates and infected the next day in triplicate with VSV-G-pseudotyped NL4.3GFP in the presence or absence of efavirenz as described above. Total DNA was isolated at the indicated time points postinfection using the QiaAmp extraction kit (Qiagen), and percentages of GFP^+^ cells were determined by flow cytometry 2 days postinfection. TaqMan qPCR was performed using GFP forward/reverse primers 5′-CAA CAG CCA CAA CGT CTA TAT CAT-3′/5′-ATG TTG TGG CGG ATC TTG AAG-3′ and the probe 6-carboxyfluorescein (FAM)–5′–CCG ACA AGC AGA AGA ACG GCA TCA A–3′–5-carboxytetramethylrhodamine (TAMRA), and for 2-LTR circle primers 5′-AAC TAG AGA TCC CTC AGA CCG AAA A-3′/5′-CTT GTC TTC GTT GGG AGT CTT AA-3′ (specific for pCSGW^++^) and probe FAM-5′-CTA GAG ATT TTC CAC ACT GAC-3′-TAMRA (95). Samples were normalized to the input total DNA concentration. TaqMan qPCRs were performed using the Applied Biosystems 7500 real-time PCR system.

### Positive selection analysis.

The sequences for SUN1 positive selection analysis were derived from the OrthoMaM website (v9.0) and aligned manually using Se-Al (http://tree.bio.ed.ac.uk/software/seal/) before subjecting the data to analysis using the HyPhy package (96) on the Datamonkey website (60, 61). For each alignment, a substitution model selection was performed and the recommended model was applied. Positive selection analysis was performed using the FEL and REL methods, with a significance level of *P* < 0.05 for FEL and a Bayes factor of >50 as the cutoff for REL. SUN1 sequences for the alignment were from Homo sapiens, Pan troglodytes, Pongo pygmaeus, Nomascus leucogenys, Papio anubis, Macaca mulatta, Callithrix jacchus, Canis familiaris, Felis catus, Myotis lucifugus, Ailuropoda melanoleuca, Equus caballus, Otolemur garnettii, Ictidomys tridecemlineatus, Mustela putorius furo, Monodelphis domestica, Loxodonta africana, Mus musculus, Rattus norvegicus, Ornithorhynchus anatinus, Chlorocebus sabaeus, Ovis aries, Dasypus novemcinctus.
